# *Syzyguim guineense* Extracts Show Antioxidant Activities and Beneficial Activities on Oxidative Stress Induced by Ferric Chloride in the Liver Homogenate

**DOI:** 10.3390/antiox3030618

**Published:** 2014-09-19

**Authors:** Constant Anatole Pieme, Joseph Ngoupayo, Claude Herve Khou-Kouz Nkoulou, Bruno Moukette Moukette, Borgia Legrand Njinkio Nono, Vicky Jocelyne Ama Moor, Jacqueline Ze Minkande, Jeanne Yonkeu Ngogang

**Affiliations:** 1 Laboratory of Biochemistry, Department of Biochemistry and Physiological Sciences; Faculty of Medicine and Biomedical Sciences, University of Yaounde I, P.O. Box 1364 Yaounde, Cameroon; E-Mails: moukette2006@yahoo.fr (B.M.M.); movicky@yahoo.fr (V.J.A.M.); jngogang@yahoo.fr (J.Y.N.); 2 Department of pharmacy, Faculty of Medicine and Biomedical Sciences, University of Yaounde I, P.O. Box 1364 Yaounde, Cameroon; E-Mails: jngoupayo@yahoo.com (J.N.); khoukouzherve@yahoo.fr (C.H.K.N.); 3 Department of Biochemistry, Faculty of Sciences; University of Yaounde I, P.O. Box 812 Yaounde, Cameroon; E-Mail: njinoborle@yahoo.fr; 4 Department of Anesthesia and Reanimation, Faculty of Medicine and Biomedical Sciences, University of Yaounde I, P.O. Box 1364 Yaounde, Cameroon; E-Mail: minkandeze@yahoo.fr

**Keywords:** antioxidant activity, phenolic content, *Syzygium guineense*, radical scavenging activity, lipid peroxidation

## Abstract

The aim of this study was to determine the *in vitro* antioxidant activity, free radical scavenging property and the beneficial effects of extracts of various parts of *Syzygium guineense* in reducing oxidative stress damage in the liver. The effects of extracts on free radicals were determined on radicals DPPH, ABTS, NO and OH followed by the antioxidant properties using Ferric Reducing Antioxidant Power assay (FRAP) and hosphomolybdenum (PPMB). The phytochemical screening of these extracts was performed by determination of the phenolic content. The oxidative damage inhibition in the liver was determined by measuring malondialdehyde (MDA) as well as the activity of the antioxidant enzymes superoxide dismutase (SOD), catalase (CAT) and peroxidase. Overall, the bark extract of the ethanol/water or methanol showed the highest radical scavenging activities against DPPH, ABTS and OH radicals compared to the other extracts. This extract also contained the highest phenolic content implying the potential contribution of phenolic compounds towards the antioxidant activities. However, the methanol extract of the root demonstrated the highest protective effects of SOD and CAT against ferric chloride while the hydro-ethanol extract of the leaves exhibited the highest inhibitory effects on lipid peroxidation. These findings suggest that antioxidant properties of *S. guineense* extracts could be attributed to phenolic compounds revealed by phytochemical studies. Thus, the present results indicate clearly that the extracts of *S. guineense* possess antioxidant properties and could serve as free radical inhibitors or scavengers, acting possibly as primary antioxidants. The antioxidant properties of the bark extract may thus sustain its various biological activities.

## 1. Introduction

Oxidative damage (OD) of cellular biomolecules such as lipids, proteins and DNA is thought to play a crucial role in the incidence of several chronic diseases [1,2,3]. It is among the major causative factors in induction of many chronic and degenerative diseases including atherosclerosis, ischemic heart disease, aging, diabetes mellitus, cancer, immunosuppression, neuro-degenerative diseases and male infertility [4]. Oxidative stress is defined as the imbalance between the pro-oxidants and antioxidants in favor of the oxidants, potentially leading to damage, while antioxidants are substances which possess free radical chain reaction breaking properties. Most living species have an efficient defense system to protect themselves against the oxidative stress induced by reactive oxygen species (ROS) [5]. These antioxidants include enzymes (superoxide dismutase, catalase, glutathione peroxidase and reductase), glutathione and vitamins (e.g. vitamin E, C), as well as many dietary components and compounds from plants [6]. Antioxidants scavenge and control the formation of free radicals thereby preventing oxidative damage to cellular components arising as a consequence of chemical reactions involving ROS [7]. Most of the antioxidants isolated from higher plants are polyphenols [8]. Evaluation of polyphenols and antioxidant activity of herbal products has become an important tool to understand the medicinal property of plants. Interest in the possible health benefits of flavonoids and other polyphenolic compounds has increased in recent years owing to their potent antioxidant and free-radical scavenging activities [9,10,11]. Recent investigations have shown that the antioxidant properties of plants could be correlated with oxidative stress defense and different human diseases including cancer, atherosclerosis and the aging process [2,3]. The antioxidants can interfere with the oxidation process by reacting with free radicals, chelating free catalytic metals and also by acting as oxygen scavengers.

*Syzygium guineense* (Myrtaceae) is an odorous species native of the wooded savannahs and tropical forests of Africa [12]. This short-trunked tree grows widely in northern Benin. Its wild, oval fruits are edible with high concentrations of Ca, Mg, Fe, K and P [13,14]. *S. guineense* is included among the African plant species that are active against malaria [15]. Its bark is used in traditional medicine to treat gasto-intestinal upsets and diarrhea [16,17,18].

In Cameroon, the wood of *S. guineense* is used as fuel for the household, for construction and for carpentry [19]. Twigs and leaves are used against hookworm and leaves against amenorrhea and madness. *S. guineense* sap yields a black dye used to color textiles. The antibacterial properties of the watery extract of *S. guineense* have been demonstrated on different strains of bacteria responsible for diarrhea [20,21]. Ethanol (EtOH) extracts of the stem bark of *S. guineense* showed molluscicidal activities and cardiovascular properties, mainly the reduction of blood pressure [22]. Antibacterial activity of triterpenes isolated from *S. guineense* has been demonstrated [23]. Other biological properties such as anti-inflammatory, analgesic and immunological activities of different part of *S. guineense* have been reported [24,25]. The chemical composition of essential oil from *S. guineense* was also investigated [26]. However, no studies reporting the *in vitro* antioxidant and protective effects of extracts of *S. guineense* have been demonstrated.

## 2. Material and Methods

### 2.1. Extraction and Preparation of Extract Solution

*Syzygium guineense* (*S. guineense*) was collected in November 2013 in the Bafia town, Mbam and Inougou Central Region of Cameroon. A sample part of the plant (leaves) was identified and deposited at the National Herbarium under the reference number 20899/SRFCam. Parts of the plant (leaves, bark and roots) of *S. guineense* were carefully cleaned with distilled water, then dried in the open air in an enclosed space away from light. The different samples were ground to a coarse powder and stored for extraction. Their powders (75 g) were soaked separately in 750 mL of solvent (water or ethanol/water v/v) for 48 h followed by filtration with Watchman No 1 filter paper (Sigma-Aldrich, Munich, Gemany). The filtrates obtained were concentrated in a rotary evaporator for hydro-alcoholic (EtOH/H_2_O) extract and dried in the oven. The extracts were kept at 4 °C for testing antioxidant properties. The extraction yield was calculated. Prior to experimentation, the solutions of the plant extracts were reconstituted using the appropriate solvent and different dilutions (25, 50, 75, 150, 300 μg/mL) of each extract.

### 2.2. Determination of the Free Radical Scavenging Potential of the Samples

#### 2.2.1. Scavenging Activity of DPPH Radical

This assay measures the free radical scavenging capacity of the investigated extracts [27]. DPPH is a molecule containing a stable free radical. In the presence of an antioxidant, which can donate an electron to DPPH, the purple color typical of free DPPH radical decays, and the absorbance change at λ = 517 nm is measured. The antioxidant activity of the plant extracts are examined on the basis of the scavenging effect on the stable DPPH free radical activity. Briefly, in 3 mL of each diluted extract or vitamin C used as standard, 1 mL of a methanol solution of DPPH 0.1 mM is added. The mixture is kept in the dark at room temperature for 30 min and the absorbance is measured at 517 nm against a blank. The following equation is used to determine the percentage of the radical scavenging activity of each extract.

Scavenging effect (%) = 100 × (*A_o_* − *A_s_*)/*A_o_* (1)

where *A_o_* is the absorbance of the blank; *A_s_* is the absorbance of the sample.

#### 2.2.2. Scavenging Effect of the ABTS^+^ Radical

ABTS assay was based on a previously described method with slight modiﬁcations [28]. ABTS radical cation (ABTS^+^) was produced by the reaction of a 7 mM ABTS solution with 2.45 mM potassium persulphate. The mixture was stored in the dark at room temperature for 12 h before use. The ABTS^+^ solution was diluted with ethanol to an absorbance of 0.70 ± 0.05 at 734 nm. After addition of 25 μL of extract sample or vitamin C (standard) to 2 mL of diluted ABTS^+ ^solution, absorbance at 734 nm was measured at exactly 6 min. The decrease in absorption was used for calculating scavenging effect values. The following equation is used to determine the percentage of the radical scavenging activity of each extract.

Scavenging effect (%) = 100 × (*A_o_* − *A_s_*)/*A_o_* (2)

where *A_o_* is the absorbance of the blank; *A_s_* is the absorbance of the sample.

#### 2.2.3. Nitric Oxide Scavenging Activity

Nitric oxide scavenging activity was determined according to the Griess Illosvoy reaction [29]. The reaction mixture contained: 10 mM sodium nitroprusside (SNP) in 0.5 M phosphate buffer, pH 7.4, and various concentrations of the tested extracts to a final volume of 3 mL. After incubation for 60 min at 37 °C, Griess reagent (α-napthyl-ethylenediamine 0.1% in water and sulphanilic acid 1% in H_3_PO_4_ 5%) was added. The pink chromophore generated during diazotization of nitrite ions with sulphanilamide and subsequent coupling with α-napthyl-ethylenediamine was measured spectrophotometrically at 540 nm. The vitamin C was used as a positive control. Nitric oxide scavenging ability (%) was calculated by using the formula:

Percentage of NO radical scavenging activity (%) = 100 × (*A_o_* − *A_s_*)/*A_o_* (3)

where *A_o_* is the absorbance of the blank; *A_s_* is the absorbance of the sample.

#### 2.2.4. Hydroxyl Radical Scavenging Activity 

The scavenging activity of the extracts on hydroxyl radical was measured according to a previously described method [30]. In 1.5 mL of each diluted extract, 60 μL of FeCl_3_ (1 mM), 90 μL of 1,10-phenanthroline (1 mM), 2.4 mL of 0.2 M phosphate buffer, pH 7.8 and 150 μL of H_2_O_2_ (0.17 M) was added respectively. The mixture was then homogenized and incubated at room temperature for 5 min. The absorbance was read at 560 nm against the blank. The percentage of the radical scavenging activity of each extract was calculated from the equation below:

Percentage of OH radical scavenging activity (%) = 100 ×(*A_o_* − *A_s_*)/*A_o_* (4)

where *A_o_* is the absorbance of the blank; *A_s_* is the absorbance of the sample.

### 2.3. Determination of the Total Antioxidant Potential of the Different Samples

#### 2.3.1. Total Antioxidant Activity by Ferric Reducing Antioxidant Power Assay (FRAP)

The FRAP was determined using a previously described method with slight modifications [31]. This test measures the reduction of ferric ion to the ferrous form in the presence of antioxidant compounds. The fresh FRAP reagent consists of 500 mL of acetate buffer (300 mM pH 3.6), 50 mL of 2,4,6-tri(2-pyridyl)-*s*-triazine (TPTZ) (10 mM), and 50 mL of FeCl_3_·6H_2_O (50 mM). The colorimetric measurement was performed at 593 nm and the reaction monitored up to 12 min on 75 μL of each extract and 2 mL of FRAP reagent. The vitamin C was used to draw a standard curve and the butylated hydroxyl toluene (BHT) was used for the comparison. The absorbance was read at 593 nm. The results were expressed as mg equivalent vitamin C/g of dried extract.

#### 2.3.2. Phosphomolybdenum Total Antioxidant Assay (PPMB)

The total antioxidant activity of extracts was evaluated by green phosphomolybdenum complex according to a described method [32]. An aliquot of 10 μL of sample solution was mixed with 1 mL of reagent solution (0.6 M sulphuric acid, 28 mM sodium phosphate and 4 mM ammonium molybdate) in a micro centrifuge tube. Tubes were incubated in a dry thermal bath at 95 °C for 90 min. After cooling, the absorbance of the mixture was measured at 695 nm against a blank. Vitamin C was used as reference to draw the standard curve and BHT was used for the comparison. The reducing capacities of the analyzed extracts were expressed as mg of vitamin C equivalents/g of dried extract.

#### 2.3.3. Reducing Power Assay

The reducing power of the extracts was determined by the method described by Oyaizu [33]. Different concentrations of extracts (20, 40, 80, 120, 240 μg/mL) in 1 mL of distilled water were mixed with 2.5 mL of phosphate buffer (0.2 M, pH 6.6) and 2.5 mL of potassium ferrocyanide (1%). The mixture was incubated at 50 °C for 20 min. Aliquots (2.5 mL) of trichloroacetic acid (10%) were added to the mixture and centrifuged at 3000 rpm for 10 min. The upper layer of the solution (2.5m L) was mixed with 2.5 mL of distilled water and 0.5 mL of FeCl_3_ (0.1%). Increased absorbance was measured at 700 nm against the blank indicating increased reducing power.

### 2.4. Determination of the Phenolic Content of the Extracts

#### 2.4.1. Total Phenol Determination

The total phenol was determined by the Folin-Ciocalteu method [34], the reaction mixture contained: 200 μL of diluted spice extract, 800 μL of freshly prepared diluted Folin-Ciocalteu reagent and 2 mL of 7.5% sodium carbonate. The final mixture was diluted to 7 mL with deionized water. The mixtures were kept in the dark at ambient conditions for 2 h to complete the reaction. The absorbance at 765 nm was measured. Caffeic acid was used as standard and the results were expressed as mg caffeic acid (CAE)/g dried extract.

#### 2.4.2. Determination of Total Flavonoid Content

Total flavonoid content was determined using aluminum chloride (AlCl_3_) according to a known method using quercetin as a standard [35]. The spice extract (0.1 mL) was added to 0.3 mL distilled water followed by 5% NaNO_2_ (0.03 mL). After 5 min at 25 °C, AlCl_3_ (0.03 mL, 10%) was added. After further 5 min, the reaction mixture was treated with 0.2 mL of 1 mM NaOH. Finally, the reaction mixture was diluted to 1 mL with water and the absorbance was measured at 510 nm. The results were expressed as mg quercetin (QE)/g of dried extract (QE/g dried extract).

#### 2.4.3. Determination of Total Flavonols

Total flavonols in the plant extracts were estimated using a known method with modifications [36]. To 2.0 mL of sample (standard), 2.0 mL of 2% AlCl_3_ ethanol and 3.0 mL (50 g/L) sodium acetate solutions were added. The absorption at 440 nm was read after 2.5 h of incubation at 20 °C. Extract samples were evaluated at a final concentration of 0.1 mg/mL. Total flavonol content was calculated as quercetin (mg/g) using the following equation *y* = 5.3911 *x* + 0.0313, *R*² = 0.9967, based on the calibration curve, where *x* was the absorbance and the concentration was expressed as the quercetin equivalent (mg/g) dried extract (QE/g dried extract).

#### 2.4.4. Phytochemical Analysis

The phytochemical analysis of the extracts was carried out to identify the presence of proteins, lipids, alkaloids, saponins, steroids, cardiac glycosides, tannins, resins and coumarins using a standard method.

### 2.5. Protective Properties of the Plant against Oxidative Damage

#### 2.5.1. Preparation of Liver Homogenate

The liver was isolated from three normal albino *Wistar* rats. The organs were weighed and 10% (w/v) homogenate was prepared in phosphate buffer (0.1 M, pH 7.4 having 0.15 M KCl) using a homogenizer at 4 °C [37]. The homogenate was centrifuged at 3000 rpm for 15 min and clear cell-free supernatant was used for the study.

#### 2.5.2. Preparation of the Pro-Oxidative Solution

The oxidant solution was prepared directly before its utilization by adding a solution of ferric chloride 100 mM to H_2_O_2_ 0.50% prepared in phosphate buffer (0.1 M, pH 7.4). This solution was used for the investigation of the protective assays on liver enzymes.

#### 2.5.3. Total Protein Content

The total protein content of the mixture of liver was measured according to the protein kit supplier methods (Human Kit-Hu102536, Boehringer Ingelheim, Ingelheim, Germany). This result was used to express the activities of the different enzymes per g of organs.

#### 2.5.4. *In vitro* Lipid Peroxidation Assay

Lipid peroxidation assay was performed by a formerly described protocol [38]. Phosphate buffer 0.58 mL (0.1 M; PH 7.4), 200 μL samples, 200 μL liver homogenate and 20 μL ferric chloride (100 mM) were combined to form a mixture which was placed in a shaking water bath for 1 h at 37 °C. Reaction was terminated by adding 1 mL TCA (10%), TBA 1 mL (0.67%) to all the tubes which were placed in boiling water bath for 20 min. Then the test tubes were moved to a crushed ice bath and were centrifuged at 3000 rpm for 10 min. Absorbance of the supernatant was checked at 535 nm and was calculated as nM of MDA tissue using a molar extinction coefficient of 1.56 × 10^5^/M.cm.

#### 2.5.5. Determination of Peroxidase Activity

Peroxidase activity was determined by the peroxidase kit supplier with modifications. A solution containing 1 mL of the oxidant solution (FeCl_2_, 100 mM) and extract or vitamin C (standard) for a final concentration of 100 μg/mL was incubated for 1 h a water bath at 37 °C. An aliquot of PBS (0.1 mL), hydrogen peroxide (50 μL), and pyrogallol solution (110 μL) were added to distilled water (625 μL) that had been earlier dispensed into an Eppendorf tube (Fisher Scientific, Wohlen, Switzerland). The plant extract (75 μL) from the mixture was added thereafter. The same reagents were always used, except for the extract which was replaced by distilled water (75 μL), for the blank, the oxidant solution for the control and vitamin C for the standard. The reaction was mixed and incubated for at least 10 min. The solution containing 100 mM, pH 6.0 PBS (40 μL) and 0.002% (v/v) diluted liver homogenate (40 μL) was added to the blank and test mixtures respectively. These were mixed, and the increase in absorbance at 420 nm was measured at every 10 s interval for 3 min using a spectrophotometer (BioMate 3S UV-Visible, Thermo Scientific™Manufacturer, Wohlen, Switzerland). One unit of peroxidase was defined as the change in absorbance/seconds/mg of protein at 420 nm.

#### 2.5.6. Determination of Catalase Activity

Prior to the test, a solution containing a mixture of 1 mL of the oxidant solution and extract or vitamin C (standard) for a final concentration of 100 μg/mL was incubated for 1h in a water bath at 37 °C. Catalase activity of plant extracts on homogenate was assayed as previously described with modifications [38]. Briefly, an aliquot of hydrogen peroxide (0.8 mL) was dispensed into an Eppendorf tube. Phosphate buffer (1.0 mL), extracted sample/vitamin C/oxidant solution (75 μL) and (0.002% v/v) diluted homogenate (125 μL) were added. The reaction mixture (0.5 mL) was dispensed into 5% dichromate reagent (1.0 mL) and vigorously shaken. The mixture was heated in a Clifton water bath for 10 min, and allowed to cool. The absorbance at 570 nm was measured spectrophotometrically. The absorbance obtained was extrapolated from a prepared standard graph. The catalase activity was thereafter expressed as Unit/min/mg of protein:

CAT((unit)/mg protein) = (*Abs*/min × 30,000 units)/(40 cm/M × mgprotein) × *df* (5)

where *df* = dilution factor, *Abs* = absorbance.

#### 2.5.7. Superoxide Dismutase (SOD) Activity

The measurement of total SOD activity was performed according to the Misra and Fridovich method with modifications similar to those on catalase assay [39]. The principle of this method is based on the inhibition of epinephrine autoxidation. A solution of 0.2 mL distilled water and 2.5 mL sodium carbonate buffer 0.05 M, pH 10.2.was added to 0.3 mL buffered epinephrine to initiate the reaction. The absorbance at 480 nm was read for 150 s at 30 s intervals against a blank made up of 2.5 mL buffer, 0.3 mL epinephrine and 0.2 mL distilled water. The following equation allowed the calculation of the SOD activity:

SOD (unit/mg protein) = SOD (units/mL/min)/protein (mg/mL) × *df* (6)

where *df* = dilution factor.

### 2.6. Statistical Analysis

The results were presented as mean ± SEM of triplicate assays. Analyses of data were conducted using the Kruskal Wallis test and Dunnett’s multiple test (SPSS program version 18.0 for Windows, IBM Corporation, New York, NY, USA). The Log probit was used to determinate the IC_50_ and the software XLstat version 7 (Addinsoft, New York, NY, USA) was used to achieve the Pearson Correlation Analysis (PCA). The differences were considered as significant at *p* < 0.05.

## 3. Results and Discussion

### 3.1. Extraction

Table 1 shows the yield of extraction of the leaves, bark and roots of *S. guineense*. The yield varies from 10.4% (roots) to 16% (bark) for EtOH/H_2_O and 5% (roots) to 6.67% (leaves or bark) for MeOH.

**Table 1:** Yield extract of *S. guineense*.

	Extracts	Yield of Extraction	
		EtOH/H_2_O	MeOH
***S. guineense***	Leaves	15.6%	6.67%
	Bark	16%	6.67%
	Roots	10.4%	5%

### 3.2. Phytochemical Extracts

The results of the phytochemical composition of *S. guineense* extracts are given in Table 2. These results show that all samples contain sugars, proteins, lipids, polyphenols, alkaloids, saponins, steroids, cardiac glycosides, flavonoids, tannins; except for the EtOH/H_2_O extract of the bark, which contains more coumarins. The results of the polyphenolic content of the extracts show the presence of total polyphenols, flavonoids and flavonols in the different parts of the *S. guineense* extracts studied (Table 3). The total polyphenol content of the extracts varies between 0.38 and 4.6 mg/g of dried material (DM) depending on type of solvent and the part of the plant. This level of polyphenol varied significantly (*p* < 0.05) between the different parts of the plant between the solvents. The result indicates that the bark has the highest level of polyphenols and that EtOH/H_2_O was the best solvent.

**Table 2:** Phytochemical composition of extracts of *S. guineense*.

Phytochemical Compounds	EtOH/H_2_O			MeOH		
	Leaves	Bark	Roots	Leaves	Bark	Roots
**Sugars**	+	+	+	+	+	+
**Proteins**	+	+	+	+	+	+
	+	+	+	+	+	+
**Lipids**	+	+	+	+	+	+
**Alkaloids**	+	+	+	+	+	+
**Saponins**	+	+	+	+	+	+
**Stéroids/terpenoids**	+	+	+	+	+	+
**Cardiac glycosides**	+	+	+	+	+	+
**Tannins**	+	+	+	+	+	+
**Resins**	−	−	−	−	−	−
**Acids**	−	−	−	−	−	−
**Coumarins**	−	+	−	−	−	−

(+) presence, (−) absence.

Regarding total flavonoids, the level varied from 0.05 to 0.79 mg/g of DM. This level of flavonoids was significantly higher (*p* < 0.05) in the bark extract in both solvents. In EtOH/H_2_O extracts, the flavonoid content ranged from 0.79 *±* 0.04 mg/g DM (bark) to 0.25 *±* 0.02 mg/g DM (root) representing 16.92% and 8.62% respectively of the total polyphenol content. While in the MeOH extracts the flavonoids content ranges between 0.58 *±* 0.04 mg/g DM (bark) and 0.05 ± 0.01 mg EQ/g DM (root) representing 13.84% (bark) and 13.16% (root) of the total polyphenol content. The extract of the bark in the EtOH/H_2_O solvent possesses the highest flavonoid content. The flanovol level represents 9.8% and 5.34% of the total flavonoid content of the root and leaf extracts respectively with EtOH/H_2_O. Concerning the MeOH extracts, the flavonol content ranged from 0.93% to 16.2% of the flavonoid content respectively for bark and roots. The EtOH/H_2_O extract of the bark has the highest content of flavonols (Table 3).

### 3.3. Results of the Antioxidant Capacity of the Extracts of S. guineense

Given the complexity of the oxidation process and the diverse nature of antioxidants, two methods (FRAP and PPMB) were used to evaluate the antioxidant activity of extracts from different parts of *S. guineense.* The results of an antioxidant capacity are shown in Table 4. Results show that the total antioxidant capacity of the extract varies 1859.26 to 9172.22 mg AAE/g DM with FRAP and 1114.29 to 8042.86 mg of vitamin C equivalent (AAE)/g DM with PPMB. The MeOH extract of the bark has greater antioxidant capacity with the FRAP method as well as with the PPMB. These values are significantly higher compared to vitamin C used as a control (Table 4). For each solvent, the extract of the bark has the highest antioxidant capacity for both methods. Correlations between the polyphenol content and antioxidant capacity showed a significant positive correlation between total polyphenol and antioxidant capacity with FRAP and PPMB methods for the MeOH extracts of the root and bark respectively (Table 5). Similarly, a strong positive correlation was also observed for the EtOH/H_2_O extracts of leaves and roots between the polyphenols and antioxidant capacity using PPMB.

**Table 3:** Phenolic content of *S. guineensis.*

Solvents	Extracts	Total polyphenols (mg of QE/gDM)	Flavonoids (mg of QE/gDM)	Flavonols (mg of QE/gDM)
**EtOH/H_2_O**	Leaves	2.59 ± 0.03 ^a^^,^*	0.35 ± 0.04 ^a^^,^*	0.0187 ± 0.003 *
	Bark	4.67 ± 0.11 ^b^^,^*	0.79 ± 0.01 ^b^^,^*	0.0200 ± 0.003 *
	Roots	2.9 ± 0.17 ^a^^,^*	0.25 ± 0.02 ^c^^,^*	0.0245 ± 0.001 *
**MeOH**	Leaves	1.6 ± 0.05 ^b^	0.06 ± 0.01 ^a^	0.0069 ± 0.002
	Bark	4.19 ± 0.05 ^a^	0.58 ± 0.04 ^b^	0.0054 ± 0.001
	Roots	0.38 ± 0.04 ^c^	0.05 ± 0.01 ^a^	0.0081 ± 0.002

The results are expressed as mean ± SD (*n* = 3); the values with different superscripts in the same solvent are significantly different (*p* < 0.05); * significant difference (*p* < 0.05) for different solvents of the same portion of the plant.

**Table 4:** Variation antioxidant capacity of extracts of *S. guineense.*

Solvents	Extracts	FRAP (mg AAE/gDM)	PPMB (mg AAE/gDM)
**EtOH/H_2_O**	Leaves	8907.4 ± 30.6 ^a^	4000 ± 157.14 ^a^^,^*^,^^α^
	Bark	9131.48 ± 84.13 ^a^	8150 ± 135.71 ^b^^,^^α^
	Roots	9009.26 ± 87.9 ^b^^,^*	5664.29 ± 64.29 ^c^^,^*^,^^α^
**MeOH**	Leaves	8850 ± 28.87 ^a^^,^^α^	1442.86 ± 71.43 ^a^^,^^α^
	Bark	9172.22 ± 78.37 ^b^^,^^α^	8042.86 ± 42.86 ^b^^,^^α^
	Roots	1859.26 ± 50.1 ^c^^,^^α^	1114.29 ± 103.02 ^a^^,^^α^
	Vit C	9009.26 ± 42.43	5964.29 ± 78.57

The results are expressed as mean ± SD (*n* = 3); the values with different superscripts in the same solvent are significantly different (*p* < 0.05); * significant difference (*p* < 0.05) for different solvents of the same portion of the plant; ^α^ significant difference (*p* < 0.05) between the extracts and vitamin C.

### 3.4. Antioxidant Activities of the Extracts of S. guieneense by Radical Scavenging

Results of the antioxidant activity by scavenging free radicals are shown in Table 6. From these results, the ability of extracts to trap radicals expressed as the IC_50_ varies, depending on the radical tested, the portion of the plant and the solvent extraction used. These results show that all IC_50 _values obtained are higher than that of vitamin C used as a control except for the hydroxyl radical. Among the extracts obtained from EtOH/H_2_O the bark has lower IC_5O_ of 5.52 g/mL, 16.25 mg/mL, 126.35 g/mL respectively for the DPPH, ABTS and OH radical. These results are similar to the extracts obtained with MeOH solvent. A significant and negative correlation is found between the polyphenol content and nitrite radical with the EtOH/H_2_O extract obtained from leaves of *S. guineense* (Table 5).

**Table 5:** Pearson correlation analysis between polyphenol content and antioxidant properties.

Solvents	Extracts	Correlation Coefficient ( *R*^2^)					
		FRAP	PPMB	DPPH	ABTS	OH	NO
**EtOH/H_2_O**	Leaves	−0.846	0.999 *	−0.839	0.696	−0.371	−0.031 *
	Bark	−0.068	−0.318	0.133	0.972	−0.445	0.869
	Roots	0.694	0.999 *	0.280	−0.567	0.863	−0.313
**MeOH**	Leaves	−0.938	0.638	−0.638	0.973	0.681	0.213
	Bark	0.425	0.999 *	0.747	−0.488	−0.981	−0.695
	Roots	0.998 *	−0.592	−0.515	−0.796	−0.750	−0.412

*p < 0.05 statistical significant.

**Table 6:** Variation of fifty percent inhibitory concentration (IC_50_) of different extracts of *S. guineense*.

Solvents		Fifty Percent Inhibitory Concentraion (μg/mL)			
		DPPH	ABTS	OH	NO
**EtOH/H_2_O**	Leaves	5.67 ± 0 ^a^^,^*^,^^α^	92.8 ± 2.01 ^a^^,^*^,^^α^	274.4 ± 5.94 ^a^^,^^α^	276.19 ± 21 ^a^^,^*
	Bark	5.52 ± 0.02 ^b^^,^^α^	16.25 ± 0.12 ^b^^,^^*^^,^^α^	126.35 ± 1.79 ^b^^,^^α^	364.56 ± 10.79 ^b^^,^^α^
	Roots	5.66 ± 0.04 ^a^^,^*^,^^α^	116.69 ± 3.61 ^c^^,^*^,^^α^	131.67 ± 2.03 ^c^^,^*^,^^α^	370.34 ± 5.96 ^b^^,^*^,^^α^
**MeOH**	Leaves	5.86 ± 0.01 ^a^^,^^α^	64.07 ± 1.25 ^a^^,^^α^	264.85 ± 11.83 ^a^^,^^α^	527.62 ± 2.25 ^a^^,^^α^
	Bark	5.55 ± 0.02 ^b^^,^^α^	14.67 ± 0.12 ^b^^,^^α^	126.61 ± 1.23 ^b^	368.35 ± 6.21 ^b^^,^^α^
	Roots	6.01 ± 0.03 ^c^^,^^α^	393.98± 22.06 ^c^^,^^α^	240.4 ± 4.29 ^a^^,^^α^	397.16 ± 8.52 ^b^^,^^α^
	Vit C	5.35 ± 0.01	12.73 ± 0.3	112.74 ± 1.73	257.74 ± 1.73

The results are expressed as mean ± SD (*n* = 3); the values with different superscripts in the same solvent are significantly different (*p* < 0.05); * significant difference (*p* < 0.05) for different solvents of the same portion of the plant; α significant difference (*p* < 0.05) between the extracts and vitamin C.

### 3.5. Protective Effect of Extracts of S. guineensis on Some Markers Involved on Oxidative Stress

The results of the protective activity of the extracts of *S. guineensis* on some biochemical markers involved in oxidative stress are shown in Table 7. These results show for both solvents that the extract of the leaves has the best protective activity against lipid peroxidation compared to the other samples and vitamin C used as a positive control. The inhibition of lipid peroxidation by the extract of leaves EtOH/H_2_O (50.82 μmol/mg of protein) is significantly higher (*p* < 0.05) than that of MeOH extract (67.67 μmol/mg of protein) of the same part of the plant, 40 fold higher than the negative control (Table 7). Extracts of *S. guineense* act differently on some enzyme markers of stress, but these values are still lower than the normal (group receiving no treatment). These results show that the extracts of MeOH and the root bark significantly protect the CAT (1.42, 1.14) and SOD (0.0193, 0.0174) against the FeCl_3_ compared to vitamin C (0.66, 0.0174).

**Table 7:** Variation of markers involved in oxidative stress.

Solvents	Parts of Plant	SOD (μmol/min/mg of Protein)	CAT (μmol/min/mg of Protein)	Peroxidase (μmol/min/mg of Protein)	MDA (μmol/mg of Protein)
**EtOH/H_2_O**	Leaves	0.0522 *±* 0.012 *	0.65 ± 0.08 *	52 ± 5.2	50.82 ± 0.66 *^,a^
	Bark	0.0291 *±* 0.0020 *	0.61 ± 0.05 *	67 ± 4.5	73.71 *±* 4.33 *^,b^
	Roots	0.0097 *±* 0.0005	0.71 ± 0.11 *	61 ± 5.7	53.7 *±* 1.52 *^,a^
**MeOH**	Leaves	0.0135 *±* 0.0034	0.98 ± 0.02 *^,a^	62 ± 5.5	67.67 *±* 3.67 *^,a^
	Bark	0.0174 *±* 0.0002	1.14 ± 0.08 *^,b^	83 *±* 5.5 *	74.43 *±* 2.64 *^,b^
	Roots	0.0193 *±* 0.0001	1.42 *±* 0.12 *^,b^^,c^	58 *±* 0.6	73.43 *±* 0.1 *^,b^
	Nor	0.1082 ± 0.0005	1.59 ± 0.15	423 *±* 49.99	74.72 *±* 4.75
	Ctrl Neg	0.0251 *±* 0.0041	0.32 *±* 0.02	52 *±* 11.4	122.95 *±* 1.09
	VitC	0.0174 *±* 0.0002	0.66 *±* 0.06 *	54 ± 0.1	70.98 *±* 1.8 *

The results are expressed as mean *±* SD (*n* = 3); the values with different superscripts in the same solvent are significantly different (*p* < 0.05); * significant difference (*p* < 0.05) for different solvents of the same portion of the plant; α significant difference (*p* < 0.05) between the extracts and vitamin C.

Natural products continue to be one of the most important sources of lead compounds for the pharmaceutical industry. Plants have been used for a long time as an alternative source of antioxidant, medicines and remedies for treating human diseases. Our study describes the phytochemical characterization of various *S. guineense* leaves, root and bark and antioxidant effects of extracts including the protective activity. The extraction yields and the content of phenols vary with the extraction solvent used. Differences in the chemical constitution were observed in phytochemical screening of the tested samples. All samples contain sugars, proteins, lipids, polyphenols, alkaloids, saponins, steroids, cardiac glycosides, flavonoids, tannins. Quantitative determination of phenols in the extracts demonstrated that the EtOH/H_2_O bark extract possesses a comparatively higher amount of phenols (Table 3). Our results confirmed that the extraction of polyphenol can be carried out with acidified methanol or ethanol solvents [40]. Studies on anthocyanin extraction demonstrated that methanol extraction was 20% more efficient than that of ethanol and 73% less efficient than water [40]. The mixture EtOH/H_2_O, more polar than the MeOH, can easily solubilize phenolic Compositae. The polarity of a solvent can be increased by adding polar co-solvents to improve the affinity and the relative power of the polar solvent to molecules [41]. Our study showed that among the two solvents used (EtOH/H_2_O, MeOH), the highest concentrations of polyphenols, flavonoids and total flavonols were obtained with an EtOH/H_2_O mixture. Phenolic compounds are important phyto-constituents and have potential effects against different diseases because of their antioxidant properties [42]. The antioxidant activity of the phenolic compounds is due primarily to their redox properties and chemical structure, which can play an important role in the chelation of transition metals and free radicals [43]. Flavonoids containing hydroxyl functional groups, are responsible for the antioxidant effects and act by scavenging or chelating. Several methods can be used to determine the *in vitro* antioxidant capacity of natural substances due to the complex composition of phytochemicals and oxidative process [44]. In our study we used two methods, the first is based on the reduction of ferric tripyridyltriazine to ferrous complex at low pH and the second on the reduction of ammonium molybdate (PPMB). All tested samples showed good antioxidant capacities. The EtOH/H_2_O extract of the bark showed a significant antioxidant activity compared to vitamin C and other extracts. This activity of the extract is related to its high phenolic content (phenols, flavonoids and flavonols) found in this part of the plant compared to other extracts. Antioxidant activity of polyphenols from natural origin occurs in various mechanisms such as the prevention of chain initiation, peroxide decomposition, the prevention of further hydrogen abstraction, trapping free radicals, reducing ability, and the binding of transition metal ions [45]. Phenolic compounds have the ability to transfer hydrogen atoms which are generally associated with the presence of reducing agents [45,46]. In addition, the number and position of the hydroxyl groups of phenolic compounds also regulate their antioxidant activity [45]. The determination of antioxidant properties of the extract through radical scavenging (DPPH, ABTS, OH and NO) is considered to be a good indication of the ability of antioxidants to divest hydrogen even though they are not biologically relevant [47]. EtOH/H_2_O and MeOH extracts of the bark showed scavenging activity significantly higher than other extracts for radical DPPH, ABTS and OH. The free radical scavenging activity of the extracts is completed by transfering electrons or hydrogen atoms through the redox properties of polyphenols [48,49].

The production of oxidants and the protection against them is intrinsic to every living cell [50,51]. To minimize oxidative damage, organisms have developed antioxidative mechanisms thought to be triggered by increased ROS production [50]. Oxidants such as ROS are taken care of by an antioxidative defense system that consists of enzymes and metabolites in all subcellular compartments [51,52,53]. In stress conditions, normal capacities of these mechanisms are insufficient; triggering cells to increase and expand, their antioxidative network is reduced. In this context, superoxide dismutase (SOD) and catalase (CAT) together constitute an important defense against reactive oxygen species (ROS). Catalase catalyzes the conversion of hydrogen peroxide to water and oxygen. Thus, catalase reduces the tissue injury by removing the H_2_O_2_. Superoxide dismutase (SOD) catalyzes the conversion of superoxide anion free radical to H_2_O_2_ through a dismutation reaction [51,54]. In our study, enzymes activities of SOD, CAT and peroxidase varied depending on the part of the plants. All activities of the CAT and peroxidase in the groups treated with the oxidant and extracts of *S. guineense* were higher than the negative group (only with oxidant) and positive group (oxidant and vitamin C) (Table 7). The exposure of liver homogenate to ferric chloride reduces the activity of these enzymes through several mechanisms through oxidative stress while the presence of extracts restored these activities through the antioxidant property. This antioxidant activity might be exerted through modulation of antioxidant enzymes in the form of oxidative stress although the possibility of the extract acting as a ROS scavenger cannot be excluded. This property is attributed to phenolic compounds such as polyphenols, flavonoids and flavonols. Studies have shown that polyphenols are also associated with the CAT activity [55].

Lipid per-oxidation is a natural metabolic process under normal aerobic conditions and it is one of the most investigated consequences of ROS action on membrane structure and function. Polyunsaturated fatty acids (PUFA), the main components of membrane lipids, are susceptible to per-oxidation. The final stable end products of per-oxidation are aldehydes which react with TBA to form thiobarbituric acid malonaldehyde adduct with an absorbance maximum at 532 nm. In our studies the presence of various *S. guineense* extracts *in vitro* led to a reduction of adducts formation indicating their lipoprotective potential. This activity could be attributed to the hydroxyl radical scavenging by phytochemicals present in the potent extracts. Moreover reducing power and chelation of metal ion could also be responsible for providing membrane protective efficacy in extracts. Phenolics are a group of non-essential dietary components and their hydrogen donating property is responsible for the inhibition of free radical induced lipid peroxidation [56,57]. Phenolic and other non-phenolic phytochemicals present in extracts might also be involved in imparting some degree of membrane protection. Different variation observed for each of the markers of oxidative stress determined can also be influenced by various factors. The results might be due to: (a) the availability of substrates for peroxidation, (b) the presence/amount of peroxidation inducers, such as ascorbate, Fe^2+^, oxygen, initiators of free radical reactions and the functioning of the electron transport chain, which serve as a source of ROS, (c) the levels of the antioxidant defense, and (d) the differences in the membrane lipid, such as fluidity and surface charge [55,58]. The beneficial effects of *S. guineense* extracts found in this study could probably be of importance for formulating dietary supplements, as well as for developing new ingredients with improved antioxidant properties from other plant sources.

## 4. Conclusions

*S. guineense* extracts exhibited significant scavenging and antioxidant properties. The protective effects identified in this study are attributed to the phenolic compounds. However, before suggesting the potential therapeutic use of *S. guineense* extracts in preventing oxidative stress, further studies will be necessary to determine the biological benefits on kidney, heart and to obtain a better understanding of the mechanism of this important protective effect.

## Abbreviations

DPPH: 2,2-diphenyl-1-picrylhydrazyl, 1,1-diphenyl-2-picrylhydrazyl radical
FRAP: Ferric Reducing Ability power
ABTS: 2,2 -Azinobis(3-ethylbenzthiazoline)-6-sulfonic acid
BHT: Butylated hydroxytoluene
Vit C: Vitamine C
MDA: Malondialdehyde
TBA: Thiobarbituric acid
SNP: Sodium nitroprusside
SNP: Sodium nitroprusside
SOD: Superoxide dismutase
